# Behavioural therapy for inter-episode bipolar symptoms: a multiple baseline case series evaluation

**DOI:** 10.1186/s40345-025-00402-w

**Published:** 2025-12-08

**Authors:** Kim Wright, Sandra Bucci, Iona Cairns, Barnaby D. Dunn, Steven Jones, Heather O’Mahen, Daniel Scott, Rod S. Taylor

**Affiliations:** 1 Department of Psychology, University of Exeter, Perry Road, Exeter, EX4 4QG UK; 2 Division of Psychology and Mental Health, School of Health Sciences, Faculty of Biology, Medicine and Health, Manchester Academic Health Science Centre, The University of Manchester, Oxford Road, Manchester, M13 9PL UK; 3 Greater Manchester Mental Health NHS Foundation Trust, Bury New Rd, Prestwich, Manchester, M25 3BL UK; 4 Division of Health Research, Lancaster University, Spectrum Centre for Mental Health Research, Lancaster, LA1 4YT UK; 5 Devon Partnership NHS Trust, Wonford House, Dryden Road, Exeter, EX2 5AF UK; 6 MRC/CSO Social and Public Health Sciences Unit & Robertson Centre for Biostatistics, School of Health and Well Being, College of Medical, Veterinary and Life Sciences, University of Glasgow, Clarice Pears Building, 90 Byres Road, Glasgow, G12 8TB UK

**Keywords:** Bipolar disorder, Behavioural activation, Psychological therapy, Bipolar mood instability

## Abstract

**Background:**

Between major affective episodes some people with bipolar disorder experience persistent low mood or mood instability. Here we report an initial evaluation of the STABILISE programme (ISRCTN19416314; registration date 01.02.23), an adaptation of individual behavioural therapy that includes concepts and techniques addressing emotion regulation designed to support people experiencing these inter-episode symptoms. This study aimed to evaluate the safety, feasibility and acceptability of the intervention and to explore whether the pattern of clinical change had potential for the intervention to be of benefit. Twelve individuals with inter-episode bipolar symptoms received the STABILISE therapy in a randomised, multiple baseline case series. Participants were randomly assigned to wait 3, 4 or 5 weeks before commencing treatment, which comprised up to 22 sessions up to 7 months. Measures of symptoms, mood lability, recovery and quality of life were completed at intake, pre-therapy, and post therapy. Participants completed weekly measures of affective symptoms over the baseline and therapy periods, and for three weeks after.

**Results:**

All 12 participants completed the therapy programme and reported high levels of satisfaction overall. No adverse events were judged to be therapy related. There was one instance of reliable deterioration on one outcome measure. Across all parameters of clinical change 9 of the 12 participants showed an overall pattern of improvement and none showed a pattern of deterioration overall.

**Conclusions:**

This study provides preliminary support for the feasibility, acceptability, safety, and clinical potential of the STABILISE therapy. Further investigation of these aspects in a larger sample and within a randomised controlled trial design is required.

**Supplementary Information:**

The online version contains supplementary material available at 10.1186/s40345-025-00402-w.

Between major episodes of mania and depression, a substantial proportion of individuals with bipolar disorder (BD) experience low mood or mood instability (inter-episode bipolar symptoms: IEBS). The polarity of IEBS tends to be predominantly depressive rather than hypomanic [1]; however, instability of mood is common [2].

Psychological interventions for people with bipolar disorder generally focus on reducing risk of relapse or on alleviation of acute depression rather than upon addressing IEBS, yet ongoing symptoms are an important treatment target for several reasons. Individuals with BDs spend around twice as long with residual symptoms as they do within acute episodes on average [1], and up to half with Bipolar I or II Disorder report experiencing these symptoms [3]. Furthermore, ongoing bipolar symptoms are associated with increased psychiatric comorbidity, distress and poorer functioning [3–7] and with risk of developing full depression and mania [8].

Some clinical trials of pharmacological agents have examined their effects on residual BD symptoms [9], yet few consider their impact on mood instability. Despite psychological therapies for individuals with BDs being valued by service users [10] and advocated within many clinical guidelines [11, 12], we do not yet have guidance on optimal psychological interventions for people with IEBS. There is evidence that psychological therapy can reduce acute and residual depressive symptoms [13]. However, the same therapy protocols do not tend to target or measure ongoing mood instability. A small number of studies have explored psychological therapy for cyclothymic disorder, which involves ongoing bipolar mood instability [14, 15], but have not definitively tested efficacy nor do they target persistent low mood. Thus, we have neither a therapy that seeks to address both low mood and mood instability, nor a therapy that has been tested across the bipolar spectrum with respect to these issues.

In answer to this, we developed the STABILISE therapy protocol. This behavioural approach integrates concepts and techniques from two therapies, Behavioural Activation (BA) and Dialectical Behavioural Therapy (DBT), that have been used successfully in similar populations to address depression and emotional instability respectively. BA is a relatively parsimonious approach for the treatment of acute unipolar depression [16]. It posits that depression is maintained by a reduction in access to environmental sources of positive, meaningful reinforcement (reward) and thus aims to reinstate access to this through scheduling behaviours, and by addressing barriers to accessing meaningful rewards. In patients with BD, two case studies report BA to have high acceptability, no safety concerns, and to be associated with promising patterns of clinical change [17, 18].

DBT was developed for individuals with borderline personality disorder [19] but has since been applied to a range of problem areas and has been found to reduce problematic anger as well as substance use and self-harm, which are often coping behaviours for emotional instability [20–22]. According to the underpinning biosocial theory, emotion dysregulation results from an interaction between a child’s emotionally sensitive temperament and an early environment that invalidates their emotional responses. DBT uses behavioural principles to understand the sequence of events maintaining current difficulties and introduces skills for changing this sequence. In BD, a small number of case series and feasibility studies have tested DBT or DBT-informed programmes [23]. The outcomes of these suggest DBT approaches are likely to be acceptable and may offer benefit for people with BD in general. To date, only one study [24] has examined a DBT-informed (group skills training) approach for people with IEBS; feedback suggested the concepts were helpful but that attendance and tailoring of the intervention could be improved through an individual delivery format.

In this randomised, multiple baseline case series of the STABILISE intervention, the objectives were to: (i) test intervention safety, feasibility and acceptability; (ii) investigate whether the pattern of change in symptoms is consistent with the potential of the intervention to decrease symptoms (depressive symptoms, mood instability) and/or increase quality of life and sense of personal recovery.^1^

We used a case series method, whereby all participants receive the intervention, and feasibility and clinical outcomes can be assessed over time. This allows collection of information on intervention safety, feasibility and acceptability as well as an initial indication of possible clinical efficacy, informing the decision to proceed to further evaluation such as a feasibility randomised controlled trial, in keeping with guidance on the development of complex interventions [25]. Our criteria for progression to a feasibility trial are given in supplementary material 1.

## Materials & methods

The study was preregistered (ISRCTN19416314; registration date 01.02.23). Informed consent was received from all participants. The study was conducted in accordance with the Declaration of Helsinki, with appropriate ethical approvals in place (U.K. Health Research Authority reference: 320627).

### Design

This study used a two-wave, randomised, multiple baseline, ABA case-series design whereby a baseline measurement period without treatment (A) was followed by a treatment phase (B), and then a second measurement period without treatment (A) (see supplementary material 2). Length of the baseline period was randomised across participants: this increases the likelihood that changes following the onset of treatment are a consequence of treatment rather non-treatment-related factors such as repetition effects or spontaneous recovery. Following guidelines on the design of multiple baseline ABA case series [26–28], we randomised participants to between 3 and 5 weeks of baseline measurement (allowing a minimum of three weekly measurements during the baseline phase, and three baseline lengths). To replicate therapy delivery across several different therapists, we aimed to recruit a sample of 12 participants across four therapists.

To allow iterative refinement of the therapy protocol we recruited participants in two cohorts three months apart (*n* = 8 and *n* = 4 respectively). Because the therapy protocol changes made between waves were minor, the two cohorts were analysed as one.

### Participants

Participants were identified from local National Health Service (NHS) primary and secondary care mental health services and general practices, and through local advertisements in the south-west of England between March and September 2023.

Inclusion criteria were: (i) aged 18 or over; (ii) meets research diagnostic criteria for Bipolar I or II Disorder, Other Specified Bipolar Disorder or Cyclothymic Disorder, according to DSM-V [29]; (iii) does not meet criteria for a current manic or severe depressive episode; (iv) has IEBS, defined as at least mild depressive symptoms (Patient Health Questionnaire [PHQ9 [30] ≥ 5) or above-average bipolar mood instability defined as ≥ 1.3 (raw score ≥ 11) on the Affective Lability Scale short form (ALS-SF [31]) depression-elation scale; (v) is willing to engage in psychological work addressing IEBS or its impact on functioning; (vi) has sufficient English to complete questionnaires without translation; and (vii) has completed the intake measures.

Exclusion criteria were: (i) current substance dependence according to ICD-11 criteria [32]; (ii) frequent and serious self-harm that could not be safely managed in a community outpatient setting; and/or (iii) currently receiving another psychological therapy for BD.

### Intervention

The following section aligns to the TIDieR framework for intervention description [33]. The STABILISE therapy was codeveloped with individuals with lived experience of BD in themselves or a family member, and with clinicians. The co-development process was informed by Experience Based Co-Design [34] whereby a group of stakeholders identify uncertainties which are then prioritised, before being addressed collectively (see supplementary material 3). This process extended before, alongside and after the case series to harness learning from the case series work, and thus not all aspects of the therapy were finalised upon commencement of the study. However, the theoretical framework, concepts and techniques were largely predetermined and were captured in a therapist manual and set of participant handouts.

In STABILISE, in accordance with a broader behavioural perspective, the goal is not to maximise activity or high-activation positive affect, but rather to help participants find a sustainable pattern and balance of activity that enables them to live well within their situation, to support them to change their situation where possible and needed, and to make changes to patterns of behaviour that lead to problems or distress. This typically entails reducing mood-driven behaviour and increasing behaviours guided by the person’s values, plans or goals. These principles apply to both depressed and (hypo)manic states.

STABILISE acknowledges that people with IEBS have often experienced many years of extreme mood states and associated problems and distress, which may have led to fear of particular emotions or mood states. Therefore, STABILISE uses concepts from emotion regulation approaches, predominantly DBT, to help patients to recognise and discriminate between different mood and emotional states and to reduce anxiety and fear about these states, while also helping them to use this information about discriminating emotional states to then optimise implementation of behavioural principles. For further information on the content of the therapy see supplementary material 4.

The therapy involved up to 22 individual therapy sessions (including around two assessment sessions) delivered over up seven months. Sessions were one hour by default but with the option for participants to agree shorter or longer (up to 75 min) session duration if needed. The acute therapy period was followed by a consolidation period whereby patients could opt to see the therapist up to three times over 6 months.

Therapy was delivered by four therapists: two clinical psychologists, one psychological therapist and one psychological wellbeing practitioner with post-qualification experience of delivering cognitive or behavioural therapy of 18, 23, 15 and 2 years respectively. They received additional training in the approach as necessary: all therapists were involved in the initial development phase of the therapy therefore additional training required was minimal. Therapists one and three treated four patients each, therapist two treated three patients and therapist four one patient.

Therapy could be delivered face-to-face, online or by telephone according to patient preference and practical constraints. Therapy was delivered within a publicly-funded research clinic within local mental health services but outside of secondary care. Patients who were also held in secondary care had access to medication reviews with a psychiatrist as part of standard care; patients held in primary care had access to their primary care physician for mental healthcare purposes including medication management.

### Outcomes

#### Safety, feasibility and acceptability 

To assess safety, rates of adverse events, serious adverse events and the number judged to be related to the therapy were monitored at research assessments and therapy sessions, and any indications of adverse events from participant communications were followed up. Adverse events were defined as receipt of non-routine assessment or treatment for a mental or physical health issue, or where the care team were contacted by the study team regarding safety concerns. We also calculated rates of reliable deterioration in the six clinical outcome measures from pre-treatment to 7-month follow-up (see below). To assess feasibility and acceptability we recorded the number of participants expressing an interest in the study, giving consent, found to be eligible, commencing treatment and completing treatment, as well as the median, mode and range of sessions attended during treatment. In addition, participants were invited to complete a brief, purpose-designed questionnaire to rate their satisfaction with the therapy, how acceptable the approach and activities were, whether they would recommend it to a friend, and overall satisfaction with the research element of the study. Ratings were on a four-point Likert scale (supplementary material 5).

#### Weekly symptom measures

Each week during the baseline, therapy and follow-up periods participants were asked to complete: the PHQ-9, a nine item self-report measure of depressive symptoms over the past two weeks with possible scores from 0 to 21; the Altman Self-Rating Mania Scale (ASRM [35]), a five item self-report measure of hypomania symptoms over the past week with possible scores from 0 to 20; and the eight item depression-elation subscale of the ALS-SF, measuring trait mood lability between depression and elation, with possible item-mean scores from 0 to 3 (raw score range 0 to 24).

#### Other outcome measures

The following measures were completed at study intake, pre-therapy and 7 month follow-up points in addition to the weekly measures: the remaining two five-item subscales of the ALS-SF measuring mood lability between anxiety and depression and between anger and euthymia respectively, each scored from 0 to 15; the Generalised Anxiety Disorder scale (GAD-7 [36]), a seven item scale which measures anxiety symptoms scored from 0 to 21; the Brief Quality of Life in Bipolar Disorder (Brief QoLBD [37], a 12 item self-report measure of disorder-specific quality of life with scores from 12 to 60; and the Bipolar Recovery Scale (BRS [38]), a 36 visual analogue scale item self-report measure of sense of personal recovery scored from 0 to 3600.

In addition, participants were invited to report on their current mood and activity 5 times daily for 14 days via a purpose-built web application (momentary assessment block [MAB]; see supplementary material 6). These MABs took place on two occasions: during the first 14 days of the baseline phase, and during the first 14 days following the end of therapy. Each administration was expected to take participants around 2–5 min to complete. The purpose of this was to ascertain the acceptability of using momentary assessment in future studies as a means of measuring mood instability. Because of the small sample and preliminary nature of the measure we did not plan to use MABS data as a secondary outcome measure. Acceptability outcomes for the MABS are reported in supplementary material 7.

### Procedure

Following initial contact with the research team and having given consent to an initial screening call, potential participants spoke with the researcher via telephone or video conferencing to discuss the study and establish whether they would be likely to meet key eligibility criteria. Those likely to be eligible and willing to continue then attended an intake assessment interview at which they gave full study consent in writing. Consenting individuals were asked to provide demographic information (age, gender, ethnicity, current financial situation). The PHQ-9 and the depression-elation scale of the ALS-SF were administered to determine eligibility in terms of current symptoms. Research diagnostic eligibility was then assessed using the mood disorders, psychosis screening and substance dependence sections of the Structured Clinical Interview for DSM-V [39], administered by a trained member of the research team. To assess for the exclusion criterion of current severe depression the Hamilton Depression Rating Scale (HDRS [40]) was administered to individuals meeting SCID-5 criteria for current major depressive episode (exclusion threshold ≥ 24). Participants eligible and willing to continue completed the GAD7, ASRM, QoLBD, remaining ALS items and BRQ.

Participants were then randomised by a researcher independent of the study to one of three wait periods (3, 4, or 5 weeks) and completed the first MAB. Starting one week after the intake assessment participants completed the weekly symptom measures (PHQ-9, ASRM and ALS depression-elation subscale) throughout the baseline period, therapy period and for 3 weeks post therapy.

At the end of their allocated wait period, participants completed the GAD7, ASRM, QoLBD, additional ALS items and BRQ (pre-therapy assessment point). Therapy then commenced on an approximately weekly basis. At the end of therapy participants were invited to complete three weeks of post-therapy monitoring during which time they continued to complete the weekly measures and were invited to take part in a semi-structured, audio-recorded interview about their experiences of the study and the therapy (findings not reported here), as well the second MAB. The timing of this assessment point varied between participants based upon how long they opted to remain in therapy. All participants were invited to complete the battery of measures used at intake and pre-treatment for a final time seven months after study intake (7-month follow-up assessment point). Participants received £20 honorarium payment at intake and each of the three follow-up points.

### Data analysis

Analysis followed a pre-specified statistical analysis plan which was publicly registered via the study ISRCTN registration page prior to recruitment of the final participant and prior to any statistical analyses taking place.

In relation to objective one, quantitative feasibility, acceptability and safety data and aggregate scores on clinical outcome measures are presented descriptively.

In relation to objective two, we considered rates of reliable change (RC) and reliable and clinically significant change (RCSC) in scores on the PHQ-9 and depression-elation subscale of the ALS-SF to be the primary means of assessing the potential of the intervention to deliver benefit. This was assessed by exploring change from the baseline period (mean score) to the 3-week post-therapy monitoring period (mean score). Where data points were missing in either period we used the mean of the remaining points for that individual. A participant was judged to have achieved reliable change when their change score from pre to post therapy either exceeded the published RC value in the literature for that measure, or was more than 1.96 x the standard error of the pre-post score difference [41]. Our strategy for identifying RC scores for our outcomes measures was to use published reliable change scores where available and widely used; where this was not the case we calculated the RC score using the best available internal consistency and SD estimate for the measure. This was operationalised as a reported estimate derived from the largest and most similar sample available in the literature. We judged that an estimate derived from our sample would not be reliable because of the small sample size (see supplementary material 8 for details of the source of each RC score/internal consistency estimate). RC scores greater than the z-score level of 1.96 were considered statistically significant at *p* < 05. We report the proportion of participants showing: (i) reliable improvement/no change/reliable deterioration in the clinical outcome measures; (ii) improvement that is both reliable and clinically significant, where clinically significant change is defined as the scoring falling below/above the established threshold for clinical significance (dependent upon whether decrease/increase in score is considered to represent clinical improvement).

We also examined week-by-week scores across the entire therapy period for the PHQ-9 and ALS-SF depression elation subscale. We used visual methods combined with descriptive statistics [42] to allow examination of overall patterns of change. Assessment of symptom scores over time for each participant for the PHQ and ALS-SF depression elation subscale was made by two independent raters who reached consensus on each pattern (improvement, deterioration, no change) and included calculation of baseline-corrected Tau-U, an effect size estimate based on the degree of overlap between data points in successive case series phases. Following Tarlow [43], any significant baseline trend was taken into account in the calculation of Tau-U.

In secondary analysis of clinical efficacy, we calculated rates of reliable and reliable and clinically significant change on the GAD-7, QoL.BD and BRQ, based on change from pre-therapy to 7 month follow-up points and on the ASRM from the baseline period to the post therapy monitoring period.

Using continuous scores on the outcome measures, effect sizes and 90 and 95% confidence intervals were computed to obtain a preliminary estimate of the potential magnitude of change on each measure from intake assessment point to 7-month follow up assessment point, and pre-therapy assessment point to 7-month assessment point.

## Results

### Sample characteristics

Table 1 displays selected demographic and clinical characteristics of the 12 participants at study intake. The sample had a median age of 41 (range 26 to 70); the majority were female. Eleven (92%) self-described as “white British” and one (8%) as “mixed white/black African” in terms of ethnicity. In terms of their perceived financial situation, 2 (17%) reported “living comfortably (on present income)”, 6 (50%) “coping” and 4 (33%) “finding it difficult”.

**Table 1:** Clinical and demographic characteristics of participants

Ppt	Gender	Research diagnosis	Current medication	Above threshold
A	F	BD2	Y	BOTH
B	F	BD1	N	PHQ9 only
C	F	BD1	Y	BOTH
D	F	BD2	N	BOTH
E	M	BD1	Y	PHQ9 only
F	M	BD1	Y	BOTH
G	F	BD1	Y	BOTH
H	F	BD1	Y	BOTH
I	M	BDOth	Y	PHQ9 only
J	F	BD1	Y	BOTH
K	F	BD1	Y	BOTH
L	F	BD2	Y	BOTH

F = female; M = male; BD1 = Bipolar I Disorder; BD2 = Bipolar 2 Disorder; BDOth = Other specified Bipolar Disorder; “current medication” refers to current use of mood stabilising, antidepressant or antipsychotic medications at study intake; “above threshold” refers to whether participant scored above study threshold on the PHQ9, ALS-DE subscale or both (BOTH) at intake

Eight participants met research diagnostic criteria for BD I, three for BD II and one for other specified BD. Ten participants were prescribed psychiatric medication at study entry. Of these, five were prescribed one class of medication (antidepressant [*n* = 3], mood stabiliser [*n* = 2]), four were prescribed two classes (mood stabiliser and antidepressant [*n* = 2], mood stabiliser and antipsychotic [*n* = 1], antidepressant and antipsychotic [*n* = 1]) and one was prescribed all three classes. Further information on clinical characteristics of the sample are given in supplementary material 9.

**Table 2:** Mean (SD) and Pre-Post effect sizes for clinical outcome measures across the study period

Measure	Mean (SD) at intake	Mean (SD) pre-treatment^a^	Mean (SD) post-treatment^b^	Mean (SD) change intake-post	Hedges g (95% CI) [90% CI]	Mean (SD) change pre-post	Hedges g^c^ (95% CI) [90% CI]
PHQ-9	14.60 (2.92)	13.41 (3.71)	10.83 (4.21)	-	-	−2.58 (3.53)	−0.68 (−1.26, −0.07) [−1.17, −0.16]
ASRM	3.00 (3.54)	2.74 (1.75)	3.49 (3.00)	-	-	0.74 (2.98)	0.23 (−0.31, 0.76) [−0.22, 0.68]
ALS-DE	1.88 (0.80)	1.58 (0.73)	1.47 (0.68)	-	-	−0.12 (0.41)	−0.26 (−0.80, 0.28) [−0.71, 0.19]
GAD-7	10.67 (4.70)	12.92 (5.57)	10.92 (5.12)	0.25 (4.00)	0.06 (−0.47, 0.58) [−0.39, 0.50]	−2.00 (3.41)	− 0.55 (−1.11, 0.04) [−1.01, −0.05]
QoL.BD	30.17 (6.12)	30.33 (7.05)	33.83 (7.30)	3.67 (7.61)	0.45 (−0.12, 1.00) [−0.03,0.91]	3.50 (5.14)	0.63 (0.03, 1.21) [0.13, 1.11]
BRQ	2045.17 (342.58)	2011.83 (423.39)	2319.50 (409.81)	274.33 (408.61)	0.62 (0.03, 1.20) [0.12, 1.10]	307.67 (421.01)	0.68 (0.07, 1.26) [0.17, 1.17]

^**a**^Pre-treatment scores for PHQ-9, ASRM & ALS-DE calculated as the mean of participant’s scores across the pre-treatment period (including intake and weekly pre-treatment scores), hence only one change score was calculated for each of these measures (column 7). ^b^Post treatment scores for PHQ-9, ASRM & ALS-DE calculated as the mean of participant’s scores across the post-treatment period (including weekly post-treatment scores). ^c^ Cohen’s d value with Hedges correction applied. Direction of clinical improvement is positive for QoL.BD and BRQ, negative for PHQ-9, ASRM, ALS-DE and GAD-7

### Objective one: intervention safety, acceptability and feasibility

#### Study recruitment and retention

**Fig. 1:**
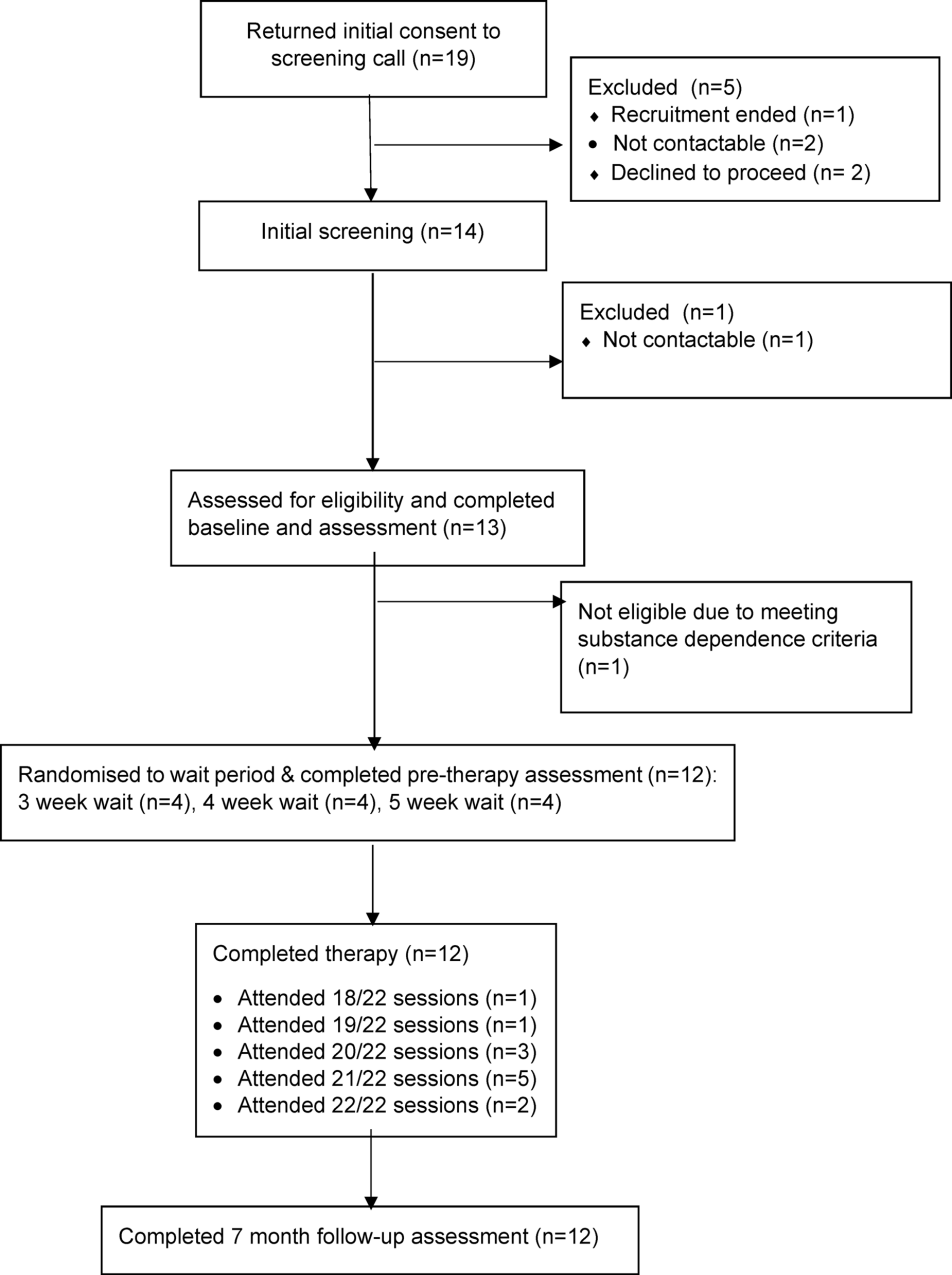
CONSORT Diagram Showing Participant Flow Through the Study

Figure 1 displays the study CONSORT flow diagram. Of 19 individuals returning consent to further contact, 14 were screened, and 13 attended the intake assessment. Of these, 12 met study entry criteria and commenced the study whilst one individual was found not to be eligible. Randomisation resulted in four participants being allocated to each of three, four or five weeks wait. All 12 participants entering the study completed the pre- and post-therapy assessment points.

#### Intervention retention and feedback

All participants attended at least 18 of the 22 possible sessions (median = 21, mode = 21). Although we did not apply a formal definition of therapy completion, none were considered to have discontinued therapy early. The total number of session cancellations was 25 (range 0–6, with 9 participants cancelling at least 1). There were two instances of a missed session without prior cancellation, both from the same participant. Of all sessions in the acute phase, 51% were in person, 36% online and 13% by telephone. Five participants used only one delivery method whilst the other seven used multiple methods.

Five participants attended all three of the optional booster sessions, three attended two booster sessions, two attended one session and two no sessions.

All participants completed the post-therapy acceptability questionnaire. Nine participants reported being “very” satisfied with the therapy, two “moderately” and one “slightly” (scale of 1 to 4), M = 3.67, SD = 0.65. Nine participants agreed “very much so” that the treatment was a good fit for their needs and the remaining three agreed “moderately”, M = 3.75, SD = 0.45. Ten participants agreed “very much so” that the therapy activities made sense, one “moderately” and one “slightly”, M = 3.75, SD = 0.62.

#### Therapy and study safety

Eight participants reported a total of 35 adverse events (15 regarding mental health, 20 physical health); none were judged by the Trial Steering Committee to be therapy related. Four were serious adverse events: two psychiatric inpatient admissions, one physical health admission and one diagnosis of a serious physical health condition. All four serious adverse events were judged to be unrelated to participation in the study or therapy. There were no reported protocol violations judged to have a significant impact on the safety of participants or the scientific integrity of the study.

There was one instance of reliable deterioration across the 12 participants, pertaining to only one of six measures (the ASRM); there was no indication it was therapy-related.

**Fig. 2:**
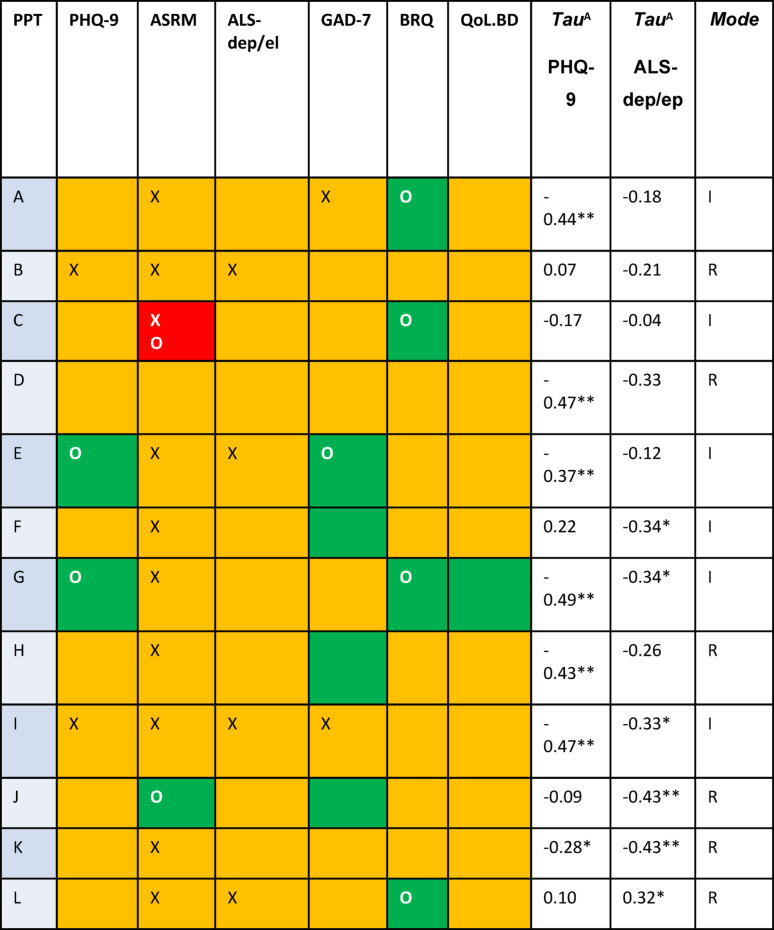
*Pattern of Reliable and Reliable and Clinically Significant Change per Participant* (*n* = 12) *Legend. X =* below clinical threshold at pre-treatment; green = reliable improvement; orange = no change; red = reliable deterioration; O = reliable and clinically significant improvement/deterioration; superscript letters after measure names depict criterion used to evaluate clinically significant change [39]: ^A^ Tau value corrected for baseline where required. * *p* <.05; **p,0.01; I = in person, R = remote.

### Objective two: pattern of change in clinical outcome measures

Table 2 displays aggregated mean scores and effect sizes for the clinical outcome measures. Figure 2 shows the pattern of reliable and reliable and clinically significant change across participants. See supplementary materials 10 for plots of week-by-week scores.

#### Rates of reliable change on clinical outcome measures

In terms of reliable change, 2/12 participants (16.7%) showed reliable improvement on the PHQ9 and none on the ALS subscale. Eight/12 participants (66.7%) showed reliable improvement on at least one of the clinical outcome measures although 4/12 (33.3%) did not show reliable change in either direction on any measure. One/12 (8.3%) participant showed reliable deterioration on one measure.

#### Pattern of change in week-by-week scores

In terms of the pattern of change in weekly scores, the Tau-U value for PHQ9 for 7/12 participants (58.3%) was negative and statistically significant (Fig. 2), suggesting improvement across the treatment phase relative to the baseline phase. Visual analysis suggested that this was due to a decrease in PHQ-9 level for all participants, with one participant also showing improvement in slope. For the ALS depression/elation subscale the Tau value for 5/12of participants (41.7%) showed a statistically significant decrease, suggesting improvement across the treatment phase relative to the baseline phase. Visual analysis suggested that this was due to a decrease in ALS level for four participants and improvement in slope for three participants. For one participant, Tau for the ALS was significantly increased indicating deterioration across the treatment phase relative to the baseline phase. Visual analysis suggested that this was due to an increasing rather than decreasing slope during the treatment phase.

#### Overall pattern of change per participant

Overall, 9/12 of participants (75%) showed a pattern of improvement on at least one parameter (six outcome measures and Tau values for PHQ-9 and ALS depression/elation subscale) and no change on the remainder. The remaining three participants showed a pattern of no change across all parameters (*n* = 1) or had an equal number of parameters showing improvement versus deterioration (*n* = 2). No participants showed an overall pattern of change characterised by deterioration. Figure 2 notes the primary therapy delivery method for each participant (in-person or remote); the small numbers and non-randomised nature of allocating therapy delivery method preclude drawing strong conclusions about the relationship between delivery method and outcome.

#### Group-level analyses of change

Table 2 shows whole-sample effect sizes for pre to post treatment change for each measure. For three of the six measures (PHQ9, BRQ, QoL.BD), the 95% confidence interval did not cross zero, was of medium size, and was in the direction of clinical improvement.

## Discussion

The findings of this study provide initial support for the feasibility, acceptability and safety of the STABILISE therapy. All participants completed the course of therapy offered with few sessions missed, and quantitative feedback indicated overall high levels of satisfaction with the therapy experience in general, the fit of the approach to participant needs, and the extent to which the therapy activities made sense. It was also found to be feasible to collect research data within the context of this study: all participants completed the post-treatment measures. With respect to therapy safety there were no serious adverse events during the study that were attributable to the intervention, meaning that our progression criterion regarding therapy safety was met.

We also aimed to investigate whether the pattern of change in symptoms shown by participants across the study period was consistent with the potential for the intervention to deliver benefit. Instances of reliable improvement on the two primary outcome measures, PHQ9 and ALS, were few. We did, however, observe significant improvement on PHQ9 at a group level, and higher rates of meaningful change on both measures in the analysis of within-person weekly scores. This may be due to ongoing variability in symptoms experienced by this population: change in overall pattern of symptoms over time may be more sensitive than comparison of a small number of data points before and after therapy.

The question of what constitutes meaningful benefit for this particular population, who by definition are experiencing ongoing issues with mood and wellbeing rather than a current acute episode and thus are less likely to show spontaneous remission, is pertinent to assessing clinical promise. The effect size for pre-post therapy depression symptom change we observed (0.68) is comparable to the generally medium-to-large pre-post effect sizes in depression symptom change observed in trials of individual psychological therapy delivered outside of acute bipolar depression [44–47], albeit with the caveat that no published trials have tested exclusively those with residual depression or ongoing mood instability. Our measure of affective instability (ALS) did not show evidence of change: it is not clear whether this is reflects lack of therapy impact on affective instability, or measurement insensitivity, a possibility given the ALS refers to trait rather than recent mood instability. Further development of robust means of measuring affective instability in trial contexts as well as evaluation of STABILISE within a randomised, controlled trial would provide a clearer picture of the potential for this approach to benefit this population.

Also on the issue of measurement it is notable that in our examination of reliable change across individuals there was often lack of congruence between improvement in symptoms and improvement in sense of personal recovery, with each being possible without the other. This supports the importance of considering sense of personal recovery as a construct distinct from symptom improvement.

Across all pre-post measures instances of reliable improvement outnumbered those of reliable deterioration, meeting our second progression criterion. Considering both the pattern of reliable change from pre- to post-treatment and the pattern of change in weekly scores, three quarters of participants showed a pattern suggestive of overall improvement while three showed a pattern of no overall change.

The STABILISE therapy includes principles and techniques from both behavioural therapy for depression and DBT. As such, our findings add to the body of literature evaluating the use of these approaches with people with BD. Previous early-stage therapy evaluation studies suggest that both approaches are acceptable and hold clinical promise [17, 18, 23]; by integrating the two the STABILISE approach enables clinicians to tailor a broadly behavioural therapy programme to be able to address both depression management and emotion regulation in the same individual.

Strengths of the study include the use of a validated, interview-based measure to ascertain research diagnosis and the use of a randomised, multiple baseline design as a means of reducing the confound between the absolute passage of time and the onset of therapy. Interpretation of week-by-week pattern of change was, however, complicated by the high degree of within-participant variability in symptom scores across both the baseline and treatment phases. To show a clear treatment effect in case series designs, it is desirable to show a stable baseline level of the outcome of interest, followed by a change after the onset of the intervention. Thus, our ability to draw conclusions regarding change in slope between baseline and treatment phases was compromised. Furthermore, as a test of clinical efficacy, studies of this nature are limited by the absence of a contemporaneous comparator group. Regarding drawing conclusions about acceptability of the therapy, our relatively small sample size and lack of diversity in terms of ethnicity limit the generalisability of our findings, as does the fact that the study was conducted at one site only, with therapy delivered by a small number of therapists. Future investigation of the STABILISE approach could address some of these limitations using a randomised, controlled trial study design with a larger sample, delivered from more than one site and by a larger number of therapists.

## Conclusion

Our findings provide preliminary support for the feasibility, acceptability, safety and potential clinical utility of the STABILISE approach. Further studies are needed to gather data on these aspects in a larger sample, and to test clinical and cost effectiveness.

## Supplementary Information

Supplementary Material 1.

Supplementary Material 2.

Supplementary Material 3.

Supplementary Material 4.

Supplementary Material 5.

Supplementary Material 6.

Supplementary Material 7.

Supplementary Material 8.

Supplementary Material 9.

Supplementary Material 10.

## Data Availability

The data presented in this study are available on request from the corresponding author provided that suitable approvals are in place and the data can be shared anonymously. The data are not publicly available due to the need to preserve the anonymity of participants.

## Abbreviations

ALS SF: Affective lability scale–short form
ASRM: Altman self-rating mania scale
BA: Behavioural activation
BD: Bipolar disorder
BRS: Bipolar recovery scale
DBT: Dialectical behavioural therapy
DSM-V: Diagnostic and statistical manual of mental disorders - fifth edition
GAD7: Generalised anxiety disorder scale – 7
HDRS: Hamilton depression rating scale
ICD-11: International classification of diseases – 11
IEBS: Interepisode bipolar symptoms
MAB: Momentary assessment block
NHS: National health service
PHQ9: Patient health questionnaire – 9
QoL.BD: Quality of life in bipolar disorder scale
RC: Reliable change
RCSC: Reliable and clinically significant change

## Footnotes

1 Findings pertaining to two additional objectives (refinement of the therapy protocol and the procedures for training and supervising therapists; development of an initial therapy competence and adherence measure) will be reported elsewhere.

## References

[1] Paykel ES, Abbott R, Morriss R, Hayhurst H, Scott J. Sub-syndromal and syndromal symptoms in the longitudinal course of bipolar disorder. Br J Psychiatry. 2006;189(2):118–23. 16880480 10.1192/bjp.bp.105.013870

[2] MacQueen GM, Marriott M, Begin H, Robb J, Joffe RT, Young LT. Subsyndromal symptoms assessed in longitudinal, prospective follow-up of a cohort of patients with bipolar disorder. Bipolar Disord. 2003;5(5):349–55. 14525555 10.1034/j.1399-5618.2003.00048.x

[3] Gershon A, Eidelman P. Inter-episode affective intensity and instability: predictors of depression and functional impairment in bipolar disorder. J Behav Ther Exp Psychiatry. 2015;46:14–8. 25164093 10.1016/j.jbtep.2014.07.005 PMC4254202

[4] Kochman FJ, Hantouche EG, Ferrari P, Lancrenon S, Bayart D, Akiskal HS. Cyclothymic temperament as a prospective predictor of bipolarity and suicidality in children and adolescents with major depressive disorder. J Affect Disord. 2005;85(1–2):181–9. 15780688 10.1016/j.jad.2003.09.009

[5] Samalin L, de Chazeron I, Vieta E, Bellivier F, Llorca P. Residual symptoms and specific functional impairments in euthymic patients with bipolar disorder. Bipolar Disord. 2016;18(2):164–73. 26946486 10.1111/bdi.12376

[6] Sperry SH, Yocum AK, McInnis MG. Mood instability metrics to stratify individuals and measure outcomes in bipolar disorder. Nat Ment Health. 2024;2(9):1111–9. 39526287 10.1038/s44220-024-00291-5 PMC11545575

[7] Stanislaus S, Faurholt-Jepsen M, Vinberg M, Coello K, Kjærstad HL, Melbye S, et al. Mood instability in patients with newly diagnosed bipolar disorder, unaffected relatives, and healthy control individuals measured daily using smartphones. J Affect Disord. 2020;271:336–44. 32479333 10.1016/j.jad.2020.03.049

[8] Judd LL, Schettler PJ, Akiskal HS, Coryell W, Leon AC, Maser JD, et al. Residual symptom recovery from major affective episodes in bipolar disorders and rapid episode relapse/recurrence. Arch Gen Psychiatry. 2008;65(4):386–94. 18391127 10.1001/archpsyc.65.4.386

[9] Alda M, McKinnon M, Blagdon R, Garnham J, MacLellan S, O’Donovan C, et al. Methylene blue treatment for residual symptoms of bipolar disorder: randomised crossover study. Br J Psychiatry. 2017;210(1):54–60. 27284082 10.1192/bjp.bp.115.173930

[10] The Bipolar Commission. Bipolar Minds Matter [Internet]. 2022 Nov. Available from: https://www.bipolaruk.org/Handlers/Download.ashx?IDMF=d4fd68a7-ffae-42bb-acf4-f7be3d903f02

[11] National Institute for Health and Care Excellence. Bipolar Disorder: Assessment and management (NICE clinical guideline CG185) [Internet]. 2014. Available from: https://www.nice.org.uk/guidance/cg185/resources/bipolar-disorder-assessment-and-management-pdf-35109814379461 31487127

[12] Yatham LN, Kennedy SH, Parikh SV, Schaffer A, Bond DJ, Frey BN, et al. Canadian network for mood and anxiety treatments (CANMAT) and international society for bipolar disorders (ISBD) 2018 guidelines for the management of patients with bipolar disorder. Bipolar Disord. 2018;20(2):97–170. 29536616 10.1111/bdi.12609 PMC5947163

[13] Yilmaz S, Huguet A, Kisely S, Rao S, Wang J, Baur K, et al. Do psychological interventions reduce symptoms of depression for patients with bipolar I or II disorder? A meta-analysis. J Affect Disord. 2022;301:193–204. 35007645 10.1016/j.jad.2021.12.112

[14] Fava GA, Rafanelli C, Tomba E, Guidi J, Grandi S. The sequential combination of cognitive behavioral treatment and well-being therapy in cyclothymic disorder. Psychother Psychosom. 2011;80(3):136–43. 21372621 10.1159/000321575

[15] Totterdell P, Kellett S, Mansell W. Cognitive behavioural therapy for cyclothymia: cognitive regulatory control as a mediator of mood change. Behav Cogn Psychother. 2012;40(4):412–24. 22353188 10.1017/S1352465812000070

[16] Richards DA, Ekers D, McMillan D, Taylor RS, Byford S, Warren FC, et al. Cost and outcome of behavioural activation versus cognitive behavioural therapy for depression (COBRA): a randomised, controlled, non-inferiority trial. Lancet. 2016;388(10047):871–80. 27461440 10.1016/S0140-6736(16)31140-0 PMC5007415

[17] Weinstock LM, Melvin C, Munroe MK, Miller IW. Adjunctive behavioral activation for the treatment of bipolar depression: a proof of concept trial. J Psychiatr Pract. 2016;22(2):149–58. 27138086 10.1097/PRA.0000000000000142 PMC4855692

[18] Wright K, Mostazir M, Bailey E, Dunn BD, O’Mahen H, Sibsey M, et al. Adapted behavioural activation for bipolar depression: a randomised multiple baseline case series. Brain Sci. 2022;12(10):1407. 36291340 10.3390/brainsci12101407 PMC9599144

[19] Linehan M. Cognitive-behavioral treatment of borderline personality disorder. Guilford Press; 1993.

[20] Ciesinski NK, Sorgi-Wilson KM, Cheung JC, Chen EY, McCloskey MS. The effect of dialectical behavior therapy on anger and aggressive behavior: a systematic review with meta-analysis. Behav Res Ther. 2022;154:104122. 35609374 10.1016/j.brat.2022.104122

[21] DeCou CR, Comtois KA, Landes SJ. Dialectical behavior therapy is effective for the treatment of suicidal behavior: a meta-analysis. Behav Ther. 2019;50(1):60–72. 30661567 10.1016/j.beth.2018.03.009

[22] Haktanır A, Callender KA. Meta-analysis of dialectical behavior therapy (DBT) for treating substance use. Res Educ Psychol. 2020;4(Special Issue):74–87.

[23] Jones BD, Umer M, Kittur ME, Finkelstein O, Xue S, Dimick MK, et al. A systematic review on the effectiveness of dialectical behavior therapy for improving mood symptoms in bipolar disorders. Int J Bipolar Disord. 2023;11(1):6. 36739574 10.1186/s40345-023-00288-6 PMC9899872

[24] Wright K, Dodd AL, Warren FC, Medina-Lara A, Dunn B, Harvey J, et al. Psychological therapy for mood instability within bipolar spectrum disorder: a randomised, controlled feasibility trial of a dialectical behaviour therapy-informed approach (the ThrIVe-B programme). Int J Bipolar Disord. 2021;9:1–13. 34195864 10.1186/s40345-021-00226-4 PMC8245616

[25] Skivington K, Matthews L, Simpson SA, Craig P, Baird J, Blazeby JM et al. A new framework for developing and evaluating complex interventions: update of Medical Research Council guidance. BMJ. 2021;n2061. 10.1136/bmj.n2061 PMC8482308 34593508

[26] Levin JR, Ferron JM. Different randomized multiple-baseline models for different situations: a practical guide for single-case intervention researchers. J Sch Psychol. 2021;86:169–77. 34051912 10.1016/j.jsp.2021.03.003

[27] Tate R, Perdices M. Quantitative data analysis for single-case methods, between-groups designs, and instrument development. Brain Impair. 2018;19(1):1–3.

[28] Kratochwill TR, Hitchcock JH, Horner RH, Levin JR, Odom SL, Rindskopf DM, et al. Single-case intervention research design standards. Remedial Spec Educ. 2013;34(1):26–38.

[29] American Psychiatric Association, editor. Diagnostic and statistical manual of mental disorders: DSM-5-TR^™^. Fifth edition, text revision. Washington, DC: American Psychiatric Association Publishing; 2022. p. 1050.

[30] Kroenke K, Spitzer RL, Williams JB. The PHQ-9: validity of a brief depression severity measure. J Gen Intern Med. 2001;16(9):606–13. 11556941 10.1046/j.1525-1497.2001.016009606.x PMC1495268

[31] Oliver MN, Simons JS. The affective lability scales: development of a short-form measure. Pers Individ Differ. 2004;37(6):1279–88.

[32] World Health Organisation. ICD-11 International Classification of Diseases (11th Revision) [Internet]. 2022. Available from: https://icd.who.int/en

[33] Hoffmann TC, Glasziou PP, Boutron I, Milne R, Perera R, Moher D, et al. Better reporting of interventions: template for intervention description and replication (TIDieR) checklist and guide. BMJ. 2014;348(mar07 3):g1687–1687. 24609605 10.1136/bmj.g1687

[34] NHS Institute for Innovation and Improvement. The EBD approach: experience based design guide and tools [Internet]. 2009. Available from: https://www.england.nhs.uk/improvement-hub/wp-content/uploads/sites/44/2017/11/Experience-Based-Design-Guide-and-Toolkit.pdf

[35] Altman EG, Hedeker D, Peterson JL, Davis JM. The Altman self-rating mania scale. Biol Psychiatry. 1997;42(10):948–55. 9359982 10.1016/S0006-3223(96)00548-3

[36] Spitzer RL, Kroenke K, Williams JB, Löwe B. A brief measure for assessing generalized anxiety disorder: the GAD-7. Arch Intern Med. 2006;166(10):1092–7. 16717171 10.1001/archinte.166.10.1092

[37] Michalak EE, Murray G. CREST. BD. Development of the QoL. BD: a disorder-specific scale to assess quality of life in bipolar disorder. Bipolar Disord. 2010;12(7):727–40. 21040290 10.1111/j.1399-5618.2010.00865.x

[38] Jones S, Mulligan LD, Higginson S, Dunn G, Morrison AP. The bipolar recovery questionnaire: psychometric properties of a quantitative measure of recovery experiences in bipolar disorder. J Affect Disord. 2013;147(1–3):34–43. 23182591 10.1016/j.jad.2012.10.003

[39] First MB, Williams JBW, Karg RS, SpitzerRL. Structured clinical interview for DSM-V disorders, clinician version (SCID-V -CV ). Arlington, VA: American Psychiatric Association.

[40] Hamilton M. A rating scale for depression. J Neurol Neurosurg Psychiatry. 1960;23(1):56. 14399272 10.1136/jnnp.23.1.56 PMC495331

[41] Ferguson RJ, Robinson AB, Splaine M. Use of the reliable change index to evaluate clinical significance in SF-36 outcomes. Qual Life Res. 2002;11:509–16. 12206571 10.1023/a:1016350431190

[42] Lane JD, Gast DL. Visual analysis in single case experimental design studies: brief review and guidelines. Neuropsychol Rehabil. 2014;24(3–4):445–63. 23883189 10.1080/09602011.2013.815636

[43] Tarlow KR. An improved rank correlation effect size statistic for single-case designs: baseline corrected Tau. Behav Modif. 2017;41(4):427–67. 27831527 10.1177/0145445516676750

[44] Ball JR, Mitchell PB, Corry JC, Skillecorn A, Smith M, Malhi GS. A randomized controlled trial of cognitive therapy for bipolar disorder: focus on long-term change. J Clin Psychiatry. 2006;67(02):277–86. 16566624 10.4088/jcp.v67n0215

[45] Lam DH, Watkins ER, Hayward P, Bright J, Wright K, Kerr N, et al. A randomized controlled study of cognitive therapy for relapse prevention for bipolar affective disorder: outcome of the first year. Arch Gen Psychiatry. 2003;60(2):145. 12578431 10.1001/archpsyc.60.2.145

[46] Cardoso TDA, Farias CDA, Mondin TC, Da Silva GDG, Souza LDDM, Da Silva RA, et al. Brief psychoeducation for bipolar disorder: impact on quality of life in young adults in a 6-month follow-up of a randomized controlled trial. Psychiatry Res. 2014;220(3):896–902. 25300245 10.1016/j.psychres.2014.09.013

[47] Isasi AG, Echeburúa E, Limiñana JM, González-Pinto A. How effective is a psychological intervention program for patients with refractory bipolar disorder? A randomized controlled trial. J Affect Disord. 2010;126(1–2):80–7. 20444503 10.1016/j.jad.2010.03.026

