# Supplementary material for: Behavioural therapy for inter-episode bipolar symptoms: a multiple baseline case series evaluation
Source: Int J Bipolar Disord. 2025 Dec 8;14:3. doi: 10.1186/s40345-025-00402-w (PMC12811185; doi:10.1186/s40345-025-00402-w)
Supplement: Supplementary file 1 — Supplementary Material 1. [file 40345_2025_402_MOESM1_ESM.docx]

**Supplementary material 1**

| Study Aspect | Criterion |
| --- | --- |
| Safety | i) no serious concerns about therapy safety (no serious adverse events that are attributable to the therapy, or if there are therapy-attributable adverse events, the independent Trial Steering Committee (TSC) are satisfied that suitable modifications have been made to the therapeutic protocol to mitigate sufficiently against future risk). |
| Potential for clinical efficacy | ii) on the outcome measures overall, number of instances of reliable improvement exceed instances of reliable deterioration. |
