# Supplementary material for: Behavioural therapy for inter-episode bipolar symptoms: a multiple baseline case series evaluation
Source: Int J Bipolar Disord. 2025 Dec 8;14:3. doi: 10.1186/s40345-025-00402-w (PMC12811185; doi:10.1186/s40345-025-00402-w)
Supplement: Supplementary file 2 — Supplementary Material 2. [file 40345_2025_402_MOESM2_ESM.docx]

**Supplementary material 2**

Schematic illustrating study design

| Wait time in weeks (randomly allocated) | Weeks | | | | | | | | | | | | | | | | | | | | | | | | | | | | | |
| --- | --- | --- | --- | --- | --- | --- | --- | --- | --- | --- | --- | --- | --- | --- | --- | --- | --- | --- | --- | --- | --- | --- | --- | --- | --- | --- | --- | --- | --- | --- |
|  | 1 | 2 | 3 | 4 | 5 | 6 | 7 | 8 | 9 | 10 | 11 | 12 | 13 | 14 | 15 | 16 | 17 | 18 | 19 | 20 | 21 | 22 | 23 | 24 | 25 | 26 | 27 | 28 | 29 | 30 |
| 3 | A |  | B |  |  |  |  |  |  |  |  |  |  |  |  |  |  |  |  |  |  |  |  |  |  | X | X | X |  | C |
| 4 | A |  |  | B |  |  |  |  |  |  |  |  |  |  |  |  |  |  |  |  |  |  |  |  |  |  | X | X | X | C |
| 5 | A |  |  |  | B |  |  |  |  |  |  |  |  |  |  |  |  |  |  |  |  |  |  |  |  |  |  | X | X | X C |

*Note.* Light shading = baseline phase; dark shading = therapy phase; X = weekly measures continue for three weeks post therapy; A = intake measures completed; B = pre-therapy measures completed; C = post-therapy measures completed.
