# Supplementary material for: Behavioural therapy for inter-episode bipolar symptoms: a multiple baseline case series evaluation
Source: Int J Bipolar Disord. 2025 Dec 8;14:3. doi: 10.1186/s40345-025-00402-w (PMC12811185; doi:10.1186/s40345-025-00402-w)
Supplement: Supplementary file 3 — Supplementary Material 3. [file 40345_2025_402_MOESM3_ESM.docx]

**Supplementary material 3**

Stakeholder Co-Development Process: Uncertainly Prioritisation

Process

1.Stakeholders on the co-design panels (individuals with lived experience of bipolar disorder in themselves or a family member, n=8; clinicians, n=2) were presented with information about the basis for the therapy, and information about the concept of developing the therapy through identifying uncertainties in therapy design and delivery.

2. The panels identified a set of uncertainties about the design and delivery of the therapy that could be addressed prior to commencing the study.

3. The panel comprising individuals with lived experience agreed an order of priority for these uncertainties.

4. Future meetings then focussed upon addressing the first and second level priority uncertainties.

Outcome

First-level priority uncertainties were identified as follows:

| Topic Area | Uncertainty | Resolution |
| --- | --- | --- |
| Structure of therapy | How do we decide how many sessions and the frequency of these? | Agreed around 2 “assessment” sessions and up to 20 therapy sessions; weekly as default but with option to have more or less frequent within a maximum 7 month period. |
|  | After the initial referral should there be a pre-therapy assessment appointment to ensure the person is in the right place to start therapy? | Not necessary in the research context; could be considered for delivery in routine care. |
|  | How should the therapist and client together decide in what order to cover different techniques, ideas, etc? | Present model and therapy modules to patient (as appropriate, avoiding overwhelming patient with information), invite patient views on which modules are of most interest. Default starting place of activity monitoring / lifestyle regulation work. |
| Engagement in therapy | How can therapy meet cultural needs? | Therapy plan and materials reviewed by external clinicians specialising in cultural factors in mental health. |
|  | How can we overcome language barriers? | Potential to translate materials using AI software; possible to have interpreter attend therapy sessions. |
|  | How / when can patient expectations be gathered and used to inform communicating realistic “outcomes” etc? | Plan to gather information from patients who receive STABILISE as part of research and use this to create personal comments / stories (with permission) to include in future iterations of materials. |
|  | How to devise a “crisis plan” / “plan b” for the therapy if someone becomes extremely unwell? | Include a therapy agreement that covers multiple aspects of therapy delivery including how therapist should notice and respond if patient experiences a crisis, misses a session or asks to cancel due to mood state. |

Second-level priorities were as follows:

| Topic Area | Uncertainty | Resolution |
| --- | --- | --- |
| Relationship with therapist | How best for the therapist to “check in” on how the patient is finding the process? | Invitation for patient feedback by therapist at each session. Review points at session 7-8 and at key points afterwards. |
|  | How do we approach care in relation to medication? | Importance of therapists allowing patient autonomy and self determination, supporting patient to make informed decisions and accessing the right advice. |
|  | How can we ask about the past in a way that feels ok? | Panel advised on wording in assessment phase in relation to asking about the past. |
|  | When and how can it be helpful to bring in examples of people living well with bipolar, or look at the positives of bipolar? | Plan to gather information from patients who receive STABILISE as part of research and use this to create personal comments / stories (with permission) to include in future iterations of materials. |
| Engagement in therapy | How can we support people to remain engaged when feeling very high or low? | Include a therapy agreement that covers multiple aspects of therapy delivery including how therapist should notice and respond if patient experiences a crisis, misses a session or asks to cancel due to mood state. Flexibility in delivery: for example sessions can be 10 minutes by telephone, or delivered during an outside walk, if patient prefers and is feasible. |
| Structure of therapy | Is there any way to bring some of the benefits of a group approach to individual work? | Hearing voices of others: see above regarding including quotes and stories from people who received the therapy. Emphasis within behavioural model on context provides opportunity to look for ways people can connect meaningfully to wider world if they wish. |
| Friends and Family | How can therapy be explained to the family / friend / supporter? | Handout for family and friends produced that patient can choose whether to share. |
|  | How can we ensure communication about the therapy with the supporters? | Agreed this is via patient; information about communication with therapist given in handout for friends and family. |
|  | Is it possible for supporters to have a relationship with the therapist? | As above. Also, patients can invite friend / family member to attend sessions if both agree. |
|  | What method could be used to encourage continuous engagement in therapy by the supporter when the motivation is no longer there? | Information about supporting your friend / family member when they are in therapy given in handout. |
| Information | How to best translate all the good work / skills / concepts / coping strategies etc from the therapy room into the patient’s real daily life? | Option of extensive pre-prepared worksheets and handouts available on a website and in a physical folder. Individualised means of retaining information (own therapy sessions, video / audio blueprint, objects, pictures). |
