# Supplementary material for: Behavioural therapy for inter-episode bipolar symptoms: a multiple baseline case series evaluation
Source: Int J Bipolar Disord. 2025 Dec 8;14:3. doi: 10.1186/s40345-025-00402-w (PMC12811185; doi:10.1186/s40345-025-00402-w)
Supplement: Supplementary file 4 — Supplementary Material 4. [file 40345_2025_402_MOESM4_ESM.docx]

**Supplementary material 4**

**Further information on the content of the STABILISE intervention**

Main components and sequencing

The STABILISE intervention follows a similar sequence to that used in Behavioural Activation for Depression (BA)^1^, whereby following initial assessment sessions participants engage in activity monitoring and scheduling. As therapy progresses, functional analysis is used to understand patterns that prevent engagement in helpful behaviours or that serve to maintain distress. Alternative behaviours are then introduced and various tools (such as problem-solving, skills training, modelling and shaping) are used to support the individual to be able to engage in these in everyday life. Additional “modules” can be introduced including a focus upon rumination, substance use, functional equivalence and anxiety. Towards the end of therapy there is typically consideration of consolidation of learning, and actions that can be taken to reduce risk of relapse. Between-session work is set collaboratively and is intended to build upon or consolidate the topic of the session or to prepare for the next session.

STABILISE differs from standard BA in the following ways:

1. A flexible approach is taken to the activity monitoring and scheduling phase, recognising that not all patients are suitably resourced or motivated to engage in the full version of this process. The principle of helping the person to follow a plan that supports their stability and wellbeing is followed, whereby the information to guide this can come from one or more sources including a mood diary but also self-report of historically helpful patterns, and knowledge of goal and value congruent activities that could be planned in. For patients where lifestyle issues are prominent or who would benefit from a very concrete means of reflecting on activities, a lifestyle “stock-take” followed by planning in of potentially helpful lifestyle behaviours can be used for this purpose.
2. Therapist awareness of the role of sleep disturbance and circadian factors in maintaining vulnerability for some people with bipolar informs the activity and lifestyle planning phase. This is done collaboratively and exploratively.
3. Functional analysis is more likely to include consideration of mood-driven, problematic appetitive behaviours, rather than only problematic avoidance behaviours.
4. Additional “modules” include a greater range of topics including interactions with others, medication issues and understanding and responding to emotions.
5. The therapeutic stance is similar to that in standard BA however with an even greater emphasis on appropriate validation and normalisation, and a dialectical approach.
6. Some patients will carry out work specifically upon on understanding and responding to emotions if they and the therapist agree this might be helpful. In addition to this, for all patients skills in recognising, understanding and responding to internal states are woven throughout the therapy course. This includes a) the option to engage in a “welcome practice” at the start of each session whereby the patient is invited to notice how they are currently feeling in their body and mind, and meet this with a brief exercise that helps them to feel ready for the hour ahead (for example, a grounding practice, a breathing practice that is typically calming in effect, a 5 minute period to “unload” current concerns). This is used to model noticing and responding to internal states, to practice particular skills, and also to use functional analysis to learn what responses might work when faced with particular internal states; b) the therapist is particularly alert to the patient’s affective responses during the session; in descriptions of recent events and in functional analyses. These opportunities are used to model noticing and relating constructively to these states, as well as making a wise choice in terms of how to respond.
7. A number of techniques and concepts from Dialectical Behaviour Therapy (DBT^2^) can be drawn upon in order to support the person to engage in behaviours that support stability and wellbeing, and are values-consistent. The most commonly used include the concept of wise, emotion and reasonable mind (states of mind), frameworks to support interpersonal interactions, and distress tolerance techniques. This aspect of STABILISE recognises however that tools to support mindful engagement, values-consistent choice-making and interpersonal effectiveness are present within a number of therapeutic approaches, not only DBT, and therapists can draw upon any of these providing that they are used within the STABILISE frame.
8. Consideration of consolidation and relapse prevention is possible at any point in the course of therapy and is given consideration throughout. Most patients will complete relapse prevention work; for some this may be early on in therapy dependent upon the person’s priorities and risk of relapse.

Integration of concepts and techniques from DBT in STABILISE

The standard DBT programme involves four modalities: group skills training, individual sessions, out-of-session skills coaching support and therapist consultation sessions. STABILISE is not a form of DBT; instead it is informed by DBT. As above, however, STABILISE recognises that tools to support mindful engagement, values-consistent choice-making and interpersonal effectiveness are present within a number of therapeutic approaches, not only DBT, and therapists can draw upon any of these providing that they are used within the STABILISE frame. STABILISE does not offer group skills training, out-of-session skills coaching or team consultation (it offers standard behavioural therapy supervision).

Schematic depicting main components and default sequencing of STABILISE intervention

 
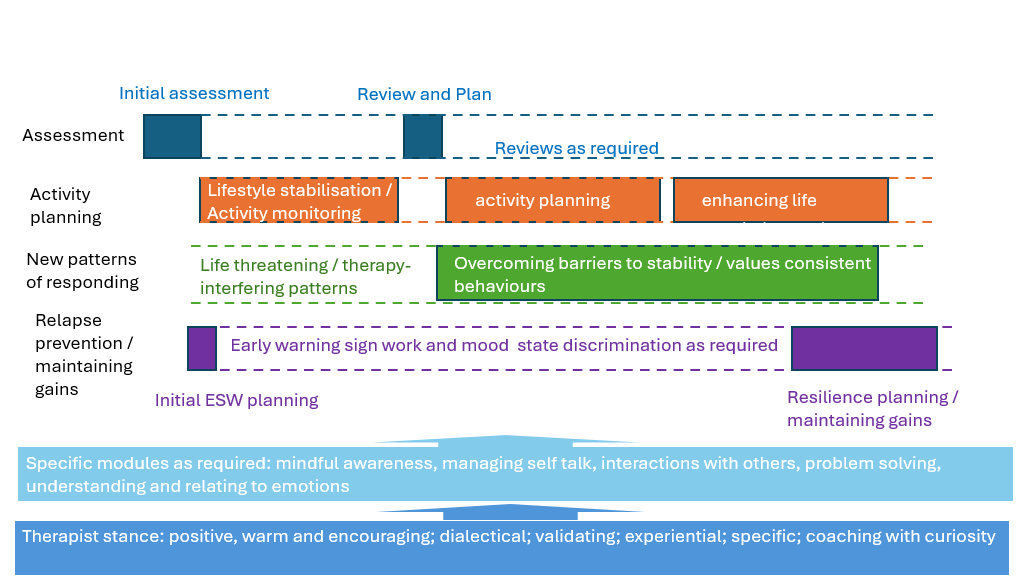


**References**

1. Richards DA, Ekers D, McMillan D, et al. Cost and Outcome of Behavioural Activation versus Cognitive Behavioural Therapy for Depression (COBRA): a randomised, controlled, non-inferiority trial. *The Lancet*. 2016;388(10047):871-880.

2. Linehan M. *Cognitive-Behavioral Treatment of Borderline Personality Disorder*. Guilford press; 1993.
