# Supplementary material for: Behavioural therapy for inter-episode bipolar symptoms: a multiple baseline case series evaluation
Source: Int J Bipolar Disord. 2025 Dec 8;14:3. doi: 10.1186/s40345-025-00402-w (PMC12811185; doi:10.1186/s40345-025-00402-w)
Supplement: Supplementary file 5 — Supplementary Material 5. [file 40345_2025_402_MOESM5_ESM.docx]

**Supplementary Material 5**

Participant Feedback Questionnaire

We would like to know what you think about the therapy you received, and your experience of the research process.

Please answer the questions below by circling the number on the scale that best describes your response. You can then expand upon each answer in the box below if you wish.

1. **not at all 2 slightly**

**3 moderately 4 very much so**

| Overall, how satisfied were you with the therapy programme? | **1 2 3 4** | |
| --- | --- | --- |
| Please add any comments about your answer here: | | |
| To what extent did the treatment approach (what the therapy was aiming to do) seem a good fit for what you were seeking help with? | **1 2 3 4** | |
| Please add any comments about your answer here: | | |
| To what extent did the activities in the therapy (the things you were invited to do in and between sessions) make sense and were reasonable? | | **1 2 3 4** |
| Please add any comments about your answer here: | | |
| How likely would you be to recommend the therapy to friends or family if they needed similar care or treatment? | **1 2 3 4** | |
| Please add any comments about your answer here: | | |
| How satisfied were you with the research aspects of the study (the research interviews and questionnaires)? | **1 2 3 4** | |
| Please add any comments about your answer here: | | |
| Please note here any other comments about the therapy or taking part in the study: | | |
