# Supplementary material for: Behavioural therapy for inter-episode bipolar symptoms: a multiple baseline case series evaluation
Source: Int J Bipolar Disord. 2025 Dec 8;14:3. doi: 10.1186/s40345-025-00402-w (PMC12811185; doi:10.1186/s40345-025-00402-w)
Supplement: Supplementary file 6 — Supplementary Material 6. [file 40345_2025_402_MOESM6_ESM.docx]

**Supplementary Material 6**

**MABS questions**

**Please complete these questions every day over the 10 day period, at the following times**

**9am, 12 noon, 3pm, 6pm, 9pm**

**Today’s date: _____________** **Time completed: __________________**

*Please rate your mood right now by placing a cross on the line below*


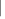

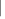

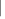

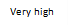

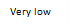


*-10                                                      0                                                       +10*

*For each item below please circle the number that best describes how you feel right now*

|  | *Not at all* |  |  | *Neutral* |  |  | *Very much* |
| --- | --- | --- | --- | --- | --- | --- | --- |
| *Right now I feel energised* | *1* | *2* | *3* | *4* | *5* | *6* | *7* |
| *Right now I feel irritable* | *1* | *2* | *3* | *4* | *5* | *6* | *7* |
| *Right now I feel empty or numb* | *1* | *2* | *3* | *4* | *5* | *6* | *7* |
| *Right now I feel restless in my mind or body* | *1* | *2* | *3* | *4* | *5* | *6* | *7* |
| *Right now I feel anxious* | *1* | *2* | *3* | *4* | *5* | *6* | *7* |
| *Right now I feel content* | *1* | *2* | *3* | *4* | *5* | *6* | *7* |
| *Right now I feel ashamed* | *1* | *2* | *3* | *4* | *5* | *6* | *7* |

*Over the past three hours, think of a significant activity you have done.*


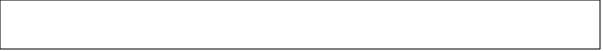
*Please write a couple of words here to name the activity*:

*How much of a sense of achievement did it give you, compared to what you expected?* Please mark the scale below.


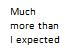

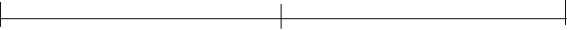

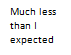


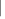

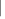

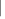
*-10                                                      0                                                       +10*

*How pleasurable was it, compared to what you expected?* Please mark the scale below.


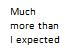

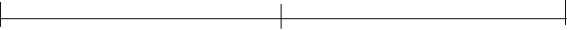

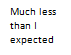


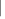

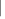

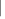
*-10                                                      0                                                       +10*

*How connected to other people did it make you feel, compared to what you expected?*


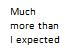

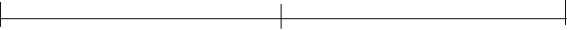

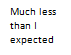


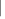

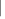

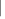
*-10                                                      0                                                       +10*

Which of these categories best describes the activity that you identified? [please tick **one** only]

**List of activity categories:**

Working / volunteering / studying

Housework / life admin / personal grooming / essential shopping

Taking care of others

Spiritual, religious, creative, cultural, time in nature

Sleeping, resting, or absence of activity

More active leisure activities

Less active leisure activities

Romantic / sexual

Healthcare activities

Thinking

Spending time with close friends / family

Wider socialising

Leisure shopping

Non-essential eating

Drinking alcohol / using drugs

 “Thrill-seeking” activities

Please select any additional categories that describe the activity [please tick as many as apply – if none apply, leave blank]

**List of activity categories:**

Working / volunteering / studying

Housework / life admin / personal grooming / essential shopping

Taking care of others

Spiritual, religious, creative, cultural, time in nature

Sleeping, resting, or absence of activity

More active leisure activities

Less active leisure activities

Romantic / sexual

Healthcare activities

Thinking

Spending time with close friends / family

Wider socialising

Leisure shopping

Non-essential eating

Drinking alcohol / using drugs

 “Thrill-seeking” activities
