# Supplementary material for: Behavioural therapy for inter-episode bipolar symptoms: a multiple baseline case series evaluation
Source: Int J Bipolar Disord. 2025 Dec 8;14:3. doi: 10.1186/s40345-025-00402-w (PMC12811185; doi:10.1186/s40345-025-00402-w)
Supplement: Supplementary file 7 — Supplementary Material 7. [file 40345_2025_402_MOESM7_ESM.docx]

**Supplementary material 7**

**Acceptability outcome data for the MABs**

The pre and post therapy momentary assessment blocks (MABs) were included to explore the feasibility of measuring mood stability using this method. Across the pre-therapy MAB the number of assessments completed across participants, out of a possible 70, ranged from 4 (6%) to 70 (100%), mean=49.42 (SD 22.22, 71%), median=60.00 (86%). Across the post-therapy period the number of assessments completed across participants ranged from 0 to 67 (96%), mean=32.50 (SD 26.08, 46%), median=23.50 (34%).
