# Supplementary material for: Behavioural therapy for inter-episode bipolar symptoms: a multiple baseline case series evaluation
Source: Int J Bipolar Disord. 2025 Dec 8;14:3. doi: 10.1186/s40345-025-00402-w (PMC12811185; doi:10.1186/s40345-025-00402-w)
Supplement: Supplementary file 8 — Supplementary Material 8. [file 40345_2025_402_MOESM8_ESM.docx]

Supplementary material 9

Further information about the study sample

When asked to estimate number of previous episodes participants reported a median of 30 depressive episodes (range 6-99, values >99 winsorised). Those with a research diagnosis of BD I reported a median of 3 previous episodes of mania (range 1-15) whilst those with a research diagnosis of BD II reported a median of 47 previous episodes of hypomania (range 28-65; not collected for those with BD I diagnosis). The median age of onset for first mood episode was 18 (range 12-31). The high rates of depression and hypomania reported by this sample (44) may reflect the method of measurement (retrospective self-report) and the inclusion of individuals with persisting residual symptoms, a risk factor for relapse.

At intake, all 12 participants scored above the eligibility threshold on the PHQ-9 and 9 participants scored above the eligibility threshold on the ALS-SF depression-elation subscale. Table 2 displays mean scores at intake on these and the other measures. Mean self-reported symptoms were in the moderate range for depression and for anxiety and the non-clinical range for hypomania. Quality of life (QoL.BD) was lower (>1 SD below mean) than in a reference sample with BD (37), whilst sense of personal recovery (BRQ) was equivalent to a reference sample (<1 SD below mean: (38)).
