# Supplementary material for: Behavioural therapy for inter-episode bipolar symptoms: a multiple baseline case series evaluation
Source: Int J Bipolar Disord. 2025 Dec 8;14:3. doi: 10.1186/s40345-025-00402-w (PMC12811185; doi:10.1186/s40345-025-00402-w)
Supplement: Supplementary file 9 — Supplementary Material 9. [file 40345_2025_402_MOESM9_ESM.docx]

**Supplementary Material 8**

Values Used to Inform Evaluation of Reliable Change and Clinical Significance

| Measure | RC value | Source of RC value | Clinical improvement value | Source of clinical significance value |
| --- | --- | --- | --- | --- |
| PHQ9 | 6 | Established RC score used in literature(1) | Score <10 | Established cut score used in literature(1) |
| GAD | 4 | Established RC score used in literature(1) | Score <8 | Established cut score used in literature(1) |
| QoL.BD | 7.35 | RC calculation utilising SD (6.43) & Cronbach’s alpha value (0.83) from comparable sample(2) | Not possible to establish a clinical threshold score | - |
| BRQ | 405.72 | RC calculation utilising SD (414.00) & Cronbach’s alpha value (0.88) from comparable sample(3) | Score change ≥200 | No established threshold score; minimum important clinical difference value has been established based on patient focus group exercise by measure developers (Prof. Steven Jones, email communication, October 3, 2019) |
| ALS-DE | 0.77 | RC calculation utilising SD (0.72) & Cronbach’s alpha value (0.85) from comparable sample(4) | Raw integer score of ≤10 | No established threshold score. Threshold based on cut score in study entry criteria. |
| ASRM | 3.96 | RC calculation utilising SD (2.70) & Cronbach’s alpha value (0.72) from comparable sample(2) | Score ≥6 | Established cut score used in literature(5) |

**Supplementary material 6: references**

1. NHS Digital. Psychological Therapies, Annual Report on the use of IAPT services: England 2015–16.

2. Ng TH, Johnson SL. Rejection Sensitivity is Associated with Quality of Life, Psychosocial Outcome, and the Course of Depression in Euthymic Patients with Bipolar I Disorder. Cogn Ther Res. 2013 Dec;37(6):1169–78.

3. Jones S, Mulligan LD, Higginson S, Dunn G, Morrison AP. The bipolar recovery questionnaire: psychometric properties of a quantitative measure of recovery experiences in bipolar disorder. Journal of Affective Disorders. 2013;147(1–3):34–43.

4. Aas M, Pedersen G, Henry C, Bjella T, Bellivier F, Leboyer M, et al. Psychometric properties of the Affective Lability Scale (54 and 18-item version) in patients with bipolar disorder, first-degree relatives, and healthy controls. Journal of Affective Disorders. 2015 Feb;172:375–80.

5. Altman EG, Hedeker D, Peterson JL, Davis JM. The Altman self-rating mania scale. Biological psychiatry. 1997;42(10):948–55.
