# Supplementary material for: Behavioural therapy for inter-episode bipolar symptoms: a multiple baseline case series evaluation
Source: Int J Bipolar Disord. 2025 Dec 8;14:3. doi: 10.1186/s40345-025-00402-w (PMC12811185; doi:10.1186/s40345-025-00402-w)
Supplement: Supplementary file 10 — Supplementary Material 10. [file 40345_2025_402_MOESM10_ESM.docx]

**Supplementary Material 10**

Weekly scores for individual participants on the ALS depression elation subscale

*Note*. Baseline phase runs from week 0 to BL5; treatment phase from week 1 for all participants, followed by three week post-treatment phase. Where data are missing within a phase, the line is extrapolated to the next data point.

Weekly scores for individual participants on the PHQ9

*Note*. Baseline phase runs from week 0 to BL5; treatment phase from week 1 for all participants, followed by three week post-treatment phase. Where data are missing within a phase, the line is extrapolated to the next data point.
